# Effects of habitat suitability for vectors, environmental factors and host characteristics on the spatial distribution of the diversity and prevalence of haemosporidians in waterbirds from three Brazilian wetlands

**DOI:** 10.1186/s13071-018-2847-z

**Published:** 2018-05-02

**Authors:** Samira Chahad-Ehlers, Angela Terumi Fushita, Gustavo Augusto Lacorte, Pamela Carla Pereira de Assis, Silvia Nassif Del Lama

**Affiliations:** 1 Departamento de Genética e Evolução, Universidade Federal de São Carlos, Rodovia Washington Luís, km 235 SP-310, São Carlos, SP 13565-905 Brazil; 2 Centro de Engenharia, Modelagem e Ciências Aplicadas Universidade Federal do ABC, Avenida dos Estados 5001, Santo André, São Paulo, 09210-580 Brazil; 3 Laboratório Biologia Molecular, Instituto Federal de Minas Gerais, Bambuí, Fazenda Varginha, Rodovia Bambuí - Medeiros, km 5, Bambuí, Minas Gerais 38900-000 Brazil

**Keywords:** Blood parasites, *Plasmodium*, *Haemoproteus*, Prevalence, Herons, Spoonbills, Parasitism

## Abstract

**Background:**

Wetlands are ecosystems in which vectors of avian haemosporidians live and reproduce and where waterbirds join to breed in colonies. Brazil has wetlands at different latitudes, which enables testing the influence of the ecological factors on the prevalence and diversity of haemosporidians. We identified avian haemosporidians in waterbird species in three wetlands and investigated the effects of vector habitat suitability, landscape and host characteristics on the diversity and prevalence of these parasites.

**Methods:**

We created a map with the probability of occurrence of avian haemosporidian vectors using maximum-entropy modelling based on references addressing species known to be vectors of haemosporidians in birds in Brazil. We determined the prevalence and diversity index of haemosporidians in the great egret (*Ardea alba*) (*n* = 129) and roseate spoonbill (*Platalea ajaja*) (*n* = 180) and compared the findings to data for the wood stork (*Mycteria americana*) (*n* = 199).

**Results:**

We report the first record of *Plasmodium* in the family Threskiornithidae: four lineages in the roseate spoonbill, which also presented one lineage of *Haemoproteu*s. In the family Ardeidae, we found three *Plasmodium* lineages in the great egret. The similar habitat suitability for vectors found in three wetlands explains the pattern of haemosporidian diversity determined for great egret and wood stork populations. Comparisons of haemosporidian diversity within each waterbird species and between regions showed a higher level in the central-western roseate spoonbill population than in the northern population (*P* = 0.021). Removing the host effect, we discussed the results obtained in terms of characteristics of the Pantanal region. Comparisons of *Plasmodium* spp. prevalence among waterbird species within the same wetland showed higher level in roseate spoonbill (74%) than those found in the great egret (21%) and wood stork (11%). Excluding the environmental effect, we interpreted result focusing host characteristics that favour infection: time required for nestlings to be covered by feathers and migratory behaviour.

**Conclusions:**

The map of habitat suitability showed that wetlands located in a 30° latitudinal range offer similar conditions for avian vectors species and diversity of haemosporidians. The lineages described in waterbirds were previously identified in birds of prey as *Plasmodium paranucleophilum*.

**Electronic supplementary material:**

The online version of this article (10.1186/s13071-018-2847-z) contains supplementary material, which is available to authorized users.

## Background

South America is the wettest continent on Earth, with wetlands covering approximately three million square kilometres of the continent [1]. In Brazil alone, wetlands account for approximately 20% of the total area of the country [2]. Wetlands are habitats in which species of waterbirds join to breed and many breed in colonies. This landscape is also a favourable habitat for the multiplication of vectors for avian haemosporidians. Parasite transmission occurs when the parasite is in the correct life stage, the birds are susceptible, the vector is present, and the environment is permissive [3]. There are several habitat characteristics that influence the occurrence of vectors, birds, and blood parasites. As insect vectors are ectothermic, they depend more on the climatic conditions of the habitat. Thus, environmental factors exert a greater influence on vectors than birds [3].

Vector species of avian haemosporidians belong to several subfamilies of the Culicidae, Ceratopogonidae and Hippoboscidae [4]. Haemosporidian sporozoites found to date in the natural populations of mosquitoes in Brazil have been restricted to a few species (Diptera: Culicidae) that use birds as a source of blood. For example, *Culex nigripalpus* and *Culex saltanensis* have been detected carrying avian *Plasmodium* in the state of Rio de Janeiro [5, 6] and, in a recent study employing the polymerase chain reaction (PCR) technique, *Mansonia titilans*, *Mansonia pseudotitilans* and *Culex* sp. were positive for *Plasmodium* in collections from the state of Minas Gerais [7]. The authors also found *Psorophora discrucians* positive for *Hemoproteus.* To date, there are no records of Ceratopogonidae and Hippoboscidae as vectors of haemosporidians in Brazil. Despite the small number of species known to be vectors of haemosporidians in birds in the country, the occurrence of these species is high and we propose an indirect approach for the investigation of the influence of ecological factors on the distribution of vector species [8, 9]. Using ecological niche modelling, one can design a map and interpret the recent distribution of populations of vectors for haemosporidians. This map can show which habitat areas are more suitable for vectors and, by comparison to the distribution of diversity and prevalence of parasites found in hosts, one can infer whether this factor explains such findings.

Representatives of three waterbird families, Ardeidae (herons), Ciconiidae (storks) and Threskiornithidae (spoonbills), are found in breeding colonies in wetlands in Brazil during the dry period, when food availability is higher than during the rainfall season. Colonies of herons and spoonbills are found in the northern, central-western and southern regions of Brazil, whereas storks are found mainly in the northern (equatorial) and central-western regions. Nestlings of these waterbirds are sparsely covered with plumage in the early stages of development, which favours insect bites. Indeed, it has been reported that these waterbirds can be infected by haemosporidians in early phases of life [10]. Reports on this group of waterbirds in the MalAvi database (Version 2.3.3, Nov. 17, 2017) [11] demonstrate the occurrence of *Haemoproteus* in birds of the family Threskiornithidae kept in captivity [12, 13]. There are reports of eleven bird species in the family Ardeidae with lineages of *Plasmodium*, two species with *Haemoproteus* and one species with *Leucocytozoon* [11]. In the family Ciconidae, *Plasmodium* and *Haemoproteus* have been described in nestlings of the wood stork (*Mycteria americana*), sampled in two Brazilian regions and one North American region [10] as well as one individual of the white stork (*Ciconia ciconia*) in Spain [14].

The annual establishment of breeding colonies of waterbirds in wetlands offers an opportunity to estimate the prevalence and diversity of haemosporidians in similar, but non-identical habitats and investigate whether different factors explain the distribution of diversity and prevalence indices. The prevalence of haemosporidians is expected to be greater in regions with larger numbers of avian hosts and vectors, and diversity is expected to be greater with generalist parasites in a highly diverse bird community [15]. Investigating parasites in samples of birds collected from several sites in Africa, Sehgal and colleagues [16] found that maximum temperature of the warmest month was the most important factor in predicting prevalence of *Plasmodium* spp. Precipitation and proximity to bodies of water were also found to be predictors of the prevalence of haemosporidians [17, 18]. Composition of the community as well as the migratory and dispersal status of host species have also been considered to represent host-host interactions that lead to high prevalence and diversity of parasites [19–21].

In this study, we investigated whether factors related to the environment, host-host interactions and host characteristics can explain the spatial distribution of haemosporidian diversity and prevalence in three bird species from wetlands located at different latitudes. First, we investigated whether habitat suitability for vectors explains the distribution of the diversity and prevalence of haemosporidians found in three waterbird species in wetlands. We created a map with the probability of occurrence of haemosporidian vectors using maximum-entropy modelling based on references addressing haemosporidian vectors that occur in Brazil. We hypothesised that the diversity and prevalence of blood parasites in wetlands are related to habitat suitability for vectors. Secondly, we compared the prevalence and diversity of the haemosporidians in the same waterbird species in different wetlands; in this case, the host effect was removed and we investigated whether the environmental effect and host-host interactions exert an influence on these parameters. We hypothesised that diversity indices would be higher among generalist haemosporidians and in regions with more host-host interactions. We expected prevalence to be higher in regions with higher temperatures and greater precipitation and in places where avian density favours host-host interactions. Thirdly, we compared diversity and prevalence of parasites among waterbird species within the same wetland; in this case, the environmental effect was removed and we investigated characteristics of the host species. We hypothesized that diversity and prevalence are higher for migratory species than for resident birds in the same area and the prevalence is lower in species for which nestlings are covered with plumage at an earlier age.

## Methods

### Study area

**Fig. 1:**
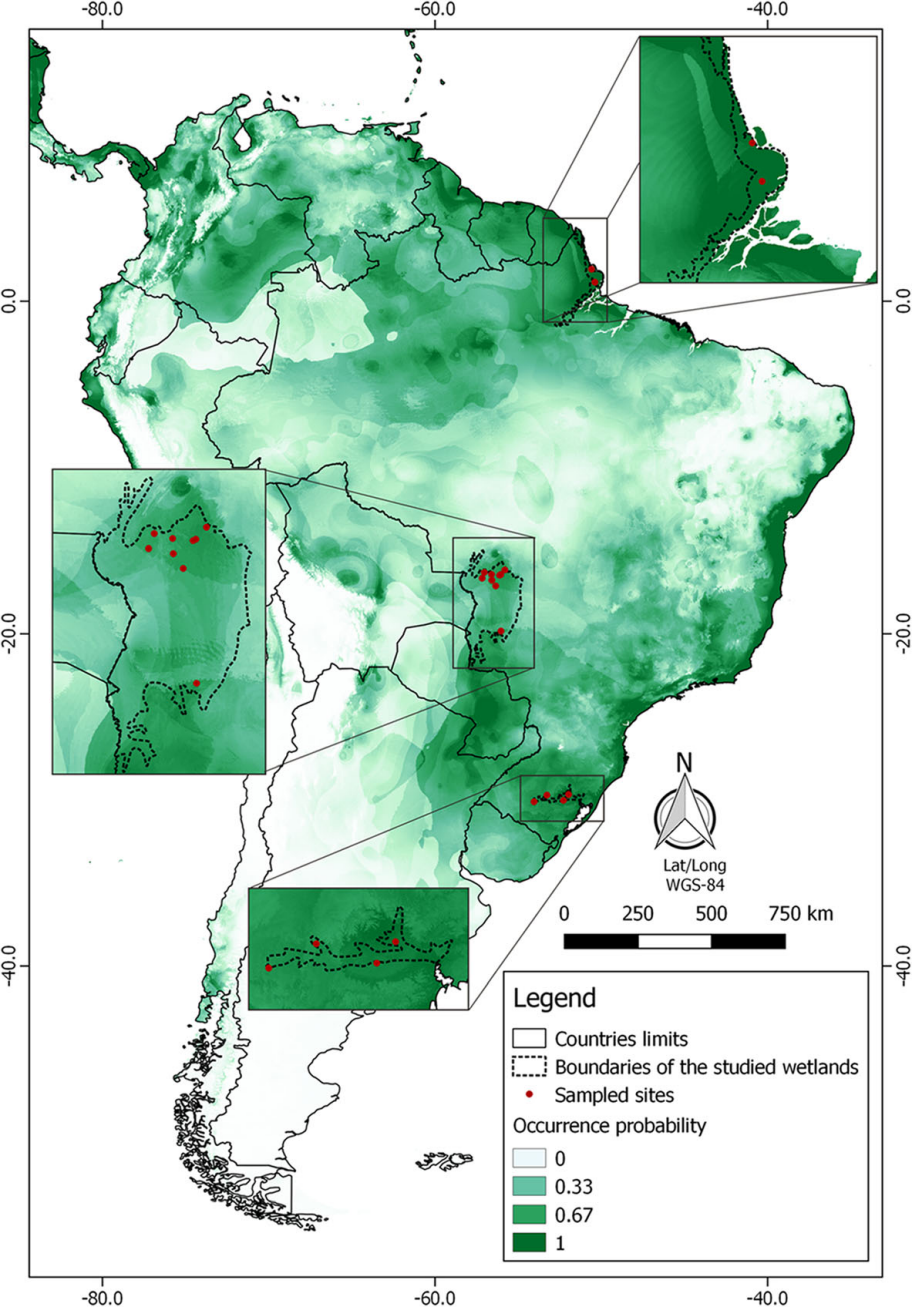
Habitat suitability for avian haemosporidian vectors in Brazil. Red points represent sampled breeding colonies of waterbirds in three wetlands areas; occurrence probability expresses niche suitability (see Additional file 1: Table S1 for geographical coordinates and habitat suitability values for each sampling site)

Blood samples were collected in breeding colonies located in three major wetlands spanning a 30° latitudinal range along Brazil: northern region (state of Amapá), central-west region (Pantanal wetland, states of Mato Grosso and Mato Grosso do Sul), and southern region (state of Rio Grande do Sul) (Fig. 1, Additional file 1: Table S1). The Brazilian Weather Bureau (INMET) offers daily weather data from 291 stations across the country. Data from 1961 onwards were digitalized and are available for download on the agency’s website [22]. The Amapá wetland has a humid tropical climate and a yearly rainfall regimen with a short dry season (three months) [23]. Bird breeding colonies were visited during the peak dry period (September-October) on temporal tidal flood plains when the mean temperature is 28.6 °C and the mean precipitation is 33.8 mm [24]. The Pantanal wetland is located in the central-western region of Brazil (138,000 km^2^) and has a warm savannah climate and a seasonal hydrological cycle [24]. Bird breeding colonies are established during the dry period and were visited in September and October, when the mean temperature is 26.7 °C and the mean precipitation is 57.1 mm [22]. The wetlands in the state of Rio Grande do Sul are located in regions of humid subtropical climate, with rains well distributed throughout the year. Waterbirds reproduce during the hot summer (January), when the mean temperature is 24.4 °C and the mean precipitation is 143.8 mm [22]. Blood samples were taken from the brachial vein of nestlings aged two to three weeks in breeding colonies; one nestling was sampled per nest (*n* = total nests). Three waterbird species were studied. Blood samples from the great egret (*Ardea alba*) (*n* = 129) and roseate spoonbill (*Platalea ajaja*) (*n* = 51) were studied herein and blood samples from the wood stork (*n* = 199) were analysed previously [10] and used in this study for the purposes of comparison.

### Ecological niche modelling

To construct the predictive maps of vectors, we first sought records of mosquito species with a preference for avian blood in the surrounding areas of the distributional survey of the birds studied. Secondly, we screened for species of vectors that have been formerly implicated in the transmission of avian haemosporidians. We selected eight species from five genera: *Aedeomyia squamipennis*, *Aedes fluviatilis*, *Anopheles albitarsis*, *Culex coronator*, *Culex nigripalpus*, *Culex quinquefasciatus*, *Culex saltanensis* and *Mansonia titilans*. These species have been mentioned as avian vectors in the available literature [5–7, 25–31] and are registered in at least one of the study areas: Amapá [32, 33]; Mato Grosso [34–36]; and Rio Grande do Sul [37–39].

Records of mosquitoes were obtained from the online databases of the Global Biodiversity Information Facility (GBIF) [40, 41] and Species Link [42]. Records of occurrence were examined for potential errors in taxonomy and distribution. Erroneous or duplicate occurrences were removed.

We modelled the potential distributions of vectors using the MaxEnt program version 3.3.3k, combining samples of location data by species and environmental variables to determine a map of occurrence with probabilities. The dataset of environmental variables was composed of proxy bioclimatic variables, which were obtained from the online public database WorldClim 1.4 at a 30-second resolution [43, 44] and included a total of 19 bioclimatic variables for the current time (1950–2000). We cropped the variables to the Neotropical region. We calculated correlations between these bioclimatic variables using Pearson’s correlation coefficient to avoid the use of significantly correlated variables (*r* > 0.85). Thus, the following variables were selected: Bio1 (annual mean temperature); Bio2 (mean diurnal range); Bio3 (isothermality); Bio7 (annual temperature range); Bio 12 (annual precipitation); Bio14 (precipitation in driest month); Bio15 (precipitation seasonality); Bio18 (precipitation in warmest quarter); and Bio19 (precipitation in coldest quarter). MaxEnt generates a “background” or “pseudo-absence” sample of the occurrence points. We used 1000 random pseudo-absences from the entire rectangular area (Neotropical region). The occurrence data for the species were separated into two sets: 75% for the model calibration/training and 25% for the model evaluation/test. The percentage contribution of each variable to the final model based on how much the variable contributed to the model dependent upon the path selected for a particular model run was provided by MaxEnt [45]. Model results were evaluated using the area under the receiver operating characteristic curve, which is commonly used to assess the performance of a MaxEnt estimate [46].

Hierarchical clustering was conducted to identify discrete groups based on the dissimilarity matrix of occurrence probability among colonies. All analyses were performed in R software [47] using the *vegan* package [48] and the commands decostant (standardise), dist (distance matrix based on Euclidean distance) and hclust (based on average).

### Detection of avian haemosporidians in blood of waterbirds

The presence of avian haemosporidians in the blood of nestlings was investigated. DNA samples extracted from the blood of waterbirds were screened for species of *Plasmodium* and *Haemoproteus* by PCR using a 478 bp fragment of the parasite *cytochome b* (*cytb*) gene from the infected individuals amplified by a nested-PCR using the primers HaemNFI (5'-CAT ATA TTA AGA GAA ITA TGG AG-3') and HaemNR3 (5'-ATA GAA AGA TAA GAA ATA-CCA TTC-3') during the first amplification and HaemF (5'-ATG GTG CTT TCG ATA TAT GCA TG-3') and HaemR2 (5'-GCA TTA TCT GGA TGT GAT AAT GGT-3') during the second amplification, following protocols described in Hellgren and colleagues [49]. After purification, the PCR products were sequenced in both directions using the BigDye Terminator Kit v.3 (Applied Biosystems, Foster City, USA) and an ABI3100® automated sequencer (Applied Biosystems). All electropherograms were aligned and edited in MEGA version 5 [50].

### Haemosporidian sequence data analyses

We considered each haplotype to be an independent lineage for the phylogenetic analyses as well as a unit of richness for diversity approaches, since there is no consensus on how to delimit haemosporidian species based on *cytb* sequences [51]. Haemosporodian genus assignment for each lineage was performed based on sequence similarity among the haplotype sequences identified in this study and sequences from the MalAvi database [11] using the BLAST analysis and assuming that the genus of the most closely related morphospecies is the most likely genus for the lineage in question.

As the number of nucleotide differences among the haemosporidian haplotypes found in aquatic birds was very small (average number of nucleotide differences among haplotypes: 2.5), the estimates of the phylogenetic relationships through conventional reconstructions (maximum likelihood or Bayesian analysis) did not generate an adequate resolution to estimate the relationship among waterbird parasite haplotypes. To increase the resolution of the relationship between *Plasmodium* haplotypes, we reconstructed haplotype networks using the median-joining network approach [52] to infer the most parsimonious solution of the haplotype network, using Network 5.0.0.1 [52]. In addition to the waterbird species haplotypes, we also included all *Plasmodium* sequences deposited in the MalAvi database with similarity ≥ 97% in the network analysis to determine whether there were previously described haplotypes with some close relationship to the waterbird haplotypes described in the present study.

Haemosporidian diversity indices were determined for each waterbird population using the DnaSP program, version 5 [53]. Diversity indices (gene or haplotype diversity [54] and nucleotide diversity [55]) were evaluated by their variances as well as using a statistical test comparing gene diversity values [56]. Prevalence was the proportion of infected birds in the total sample analysed and was calculated for each waterbird species and region. Prevalence values by species and regions were compared using Fisher’s exact test with a 5% level of significance (*P* < 0.05). These analyses were performed with the aid of the free R software [47].

## Results

### Habitat suitability for vectors

Figure 1 shows a map of Brazilian regions with differences in habitat suitability for haemosporidian vectors. The three regions studied had similar habitat suitability for vectors. Mean and standard deviation values of niche suitability were 0.656 ± 0.097 for the northern region, 0.529 ± 0.035 for the central-western region and 0.570 ± 0.020 for the southern region, with no statistically significant differences among the regions (Additional file 1: Table S1). Additional file 2: Figure S1 shows a dendrogram that also reveals no significant differences in habitat suitability for vectors among the northern, central-western, and southern regions.

### Diversity of haemosporidians in waterbird species in wetlands

The parasite haplotypes found in the waterbirds were closely related to the other *Plasmodium* sequences deposited in MalAvi using the nucleotide BLAST approach, which allowed us to assign these haplotypes as representatives of *Plasmodium* lineages. This comparative approach also revealed three new haplotypes belonging to the genus *Plasmodium* (denominated here as PLATAJP1, PLATAJP2 and PLATAJP3) (Table 1), one new haplotype belonging to the genus *Haemoproteus* (PLATAJH1) in the roseate spoonbill and two new haplotypes belonging to the genus *Plasmodium* (ARDALP1 and ARDALP2) (Table 1) in the great egret. *Plasmodium* in natural populations of the roseate spoonbill and the great egret were found in the present study, as previously reported in the wood stork [10]. *Haemoproteus* occurred in the roseate spoonbill and wood stork, but was restricted to the wetlands in the northern region of Brazil. No *Leucocytozoon* was found in the blood of the three waterbird species sampled in the three regions.

**Table 1:** Diversity indices for *Plasmodium* spp. found in three waterbird species from wetlands in Brazil

Species	Wetland region	No. infected	Diversity index				Haplotype
			s	h	H ± SD	π ± SD	
Great egret	Central-west	9	4	3	0.417 ± 0.191	0.00186 ± 0.00092	MYCAMP2, ARDALP1^a^, ARDALP2^a^
	South	12	0	1	0.000	0.000	MYCAMP2
Roseate spoonbill	North	2	0	1	0.000	0.000	MYCAMP2
	Central-west	17	5	4	0.419 ± 0.141	0.00145 ± 0.00068	MYCAMP2, PLATAJP1^a^, PLATAJP2^a^, PLATAJP3^a^
Wood stork^b^	North	6	2	3	0.600 ± 0.215	0.00139 ± 0.00058	MYCAMP2, MYCAMP3, MYCAMP7
	Central-west	14	4	5	0.505 ± 0.158	0.00120 ± 0.00044	MYCAMP1, MYCAMP2, MYCAMP4, MYCAMP5, MYCAMP6

^a^New haplotypes of *Plasmodium* described in this study. ^b^ Data reported in Villar et al. [10]

*Abbreviations*: *s* number of segregating sites, *h* number of haplotypes, *H* gene diversity [55]; *π* nucleotide diversity [56]; *SD* standard deviation

**Fig. 2:**
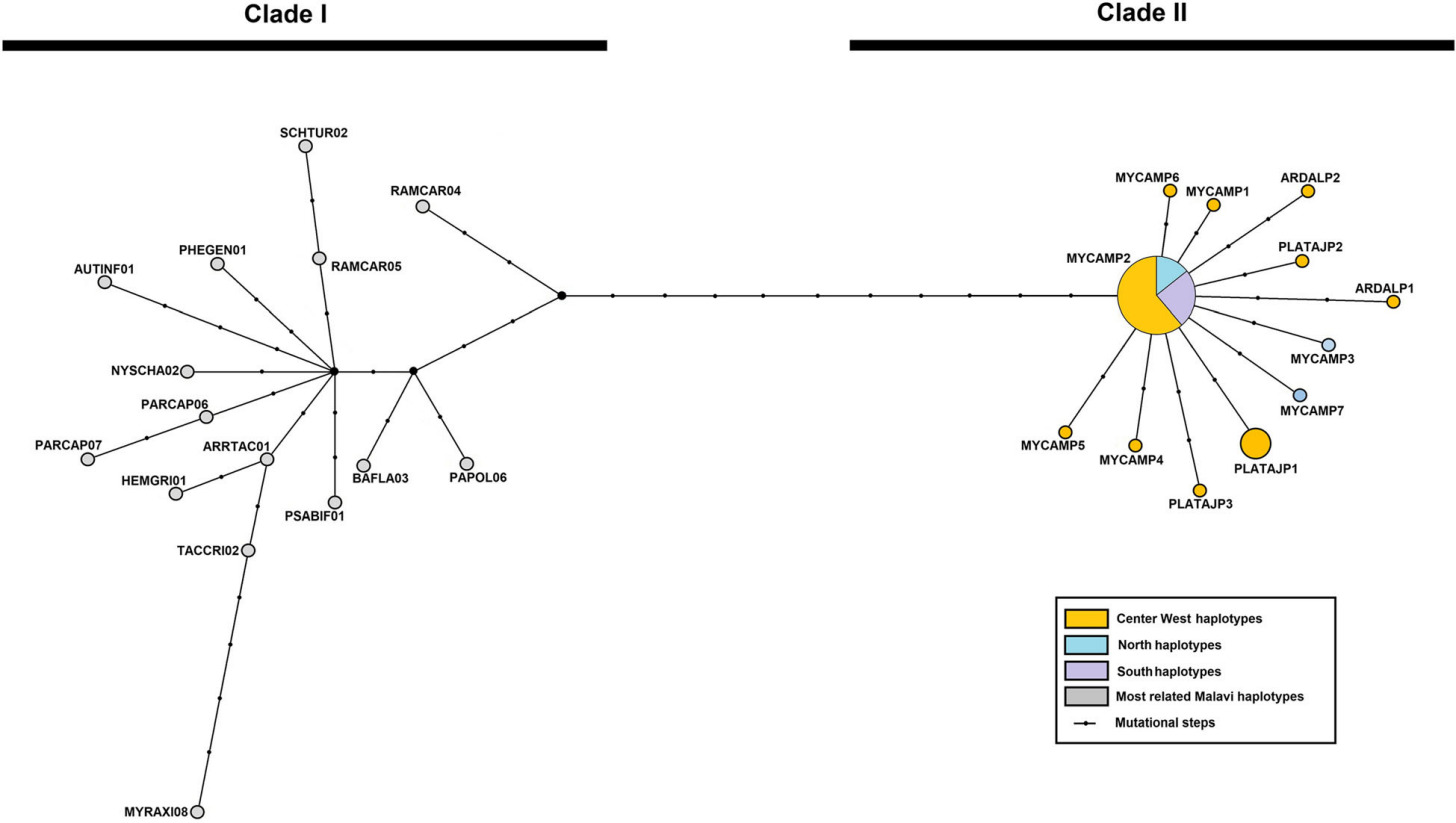
Haplotype network showing relationships between haplotypes of haemosporodians found in waterbird species studied and other species in MalAvi database. All *Plasmodium* sequences deposited in MalAvi database with similarity ≥ 97% were included in this analysis (Additional file 1: Table S2)

Haemosporidian sequence data showed that all new haplotypes found in the roseate spoonbill and great egret joined with haplotypes previously described for the wood stork in clade II, which is differentiated from previously described lineages (clade I) in the haplotype network (Fig. 2) (Additional file 1: Table S2 shows sequences deposited in the MalAvi database with similarity ≥ 97% used in the network analysis). The waterbird haplotype clade II clearly exhibits a star-like structure in which several new haplotypes (different by a singleton) derive from a high-frequency haplotype (MYCAMP2). This haplotype MYCAMP2 of *Plasmodium* was also the most prevalent in the roseate spoonbill and great egret in all regions studied, as previously identified in the wood stork [10]. This finding indicates MYCAMP2 as the ancestral haplotype, according to the network (Fig. 2, Table 1).

Comparisons of haemosporidian diversity within each waterbird species between regions showed similar levels in the wood stork and great egret populations, whereas the level of haemosporidian gene diversity was higher in the central-western roseate spoonbill population than the northern population (*P* = 0.021) (Table 1, Additional file 3: Figure S2). Comparing different bird species within each region, we found no differences in the diversity level of haemosporidians among the great egret, roseate spoonbill and wood stork in the central-western region or between the roseate spoonbill and wood stork populations in the northern region.

### Prevalence of haemosporidians in waterbirds and wetlands

Prevalences were determined for roseate spoonbill nestlings sampled from six colonies in the central-western region and two colonies in the northern region as well as for great egret nestlings from four colonies in the central-western region and four colonies in the southern region. These prevalences were compared to those found for the wood stork [10] using Fisher’s exact test (Table 2).

**Table 2:** Prevalence of haemosporidians in three waterbirds from wetlands in Brazil. Birds were infected by *Plasmodium* spp. and *Haemoproteus* spp.

Region	Colony	Wood stork^a^			Roseate spoonbill			Great egret		
		*n*	Infected by *Plas*	Infected by *Haem*	*n*	Infected by *Plas*	Infected by *Haem*	*n*	Infected by *Plas*	Infected by *Haem*
North (Amapa)	MC	29	5	0	–	–	–	–	–	–
	FA	9	0	0	–	–	–	–	–	–
	FZ	16	0	0	11	0	0	–	–	–
	SC	20	1	1	17	2	1	–	–	–
	Total	74	6	1	28	2	1			
	Mean	–	0.08	0.01	–	0.07	0.04			
Central-west (Pantanal)	BB	16	1	0	–	–	–	–	–	–
	TC	16	3	0	–	–	–	–	–	–
	RV	15	0	0	–	–	–	–	–	–
	PF	25	6	0	–	–	–	–	–	–
	FR	16	1	0	10	6	0	–	–	–
	BG	22	0	0	2	1	0	–	–	–
	FI	15	3	0	6	5	0	–	–	–
	MI	–	–	–	1	1	0	–	–	–
	RP	–	–	–	2	2	0	–	–	–
	PR	–	–	–	2	2	0	–	–	–
	TC	–	–	–	–	–	–	8	3	0
	PF	–	–	–	–	–	–	15	0	0
	PR	–	–	–	–	–	–	19	5	0
	CM	–	–	–	–	–	–	2	1	0
	Total	125	14		23	17		42	9	
	Mean	-	0.11		–	0.74		–	0.21	
South (Rio Grande do Sul)	SM	–	–	–	–	–	–	33	5	0
	SE	–	–	–	–	–	–	20	0	0
	MA	–	–	–	–	–	–	22	4	0
	PG	–	–	–	–	–	–	12	3	0
	Total							87	12	
	Mean							–	0.14	

^a^Data reported in Villar et al. [10]

*Abbreviations*: *Plas Plasmodium* spp, *Haem Haemoproteus* spp., *BB* Baía Bonita, *BG* Baía de Gaíva, *CM* Campo do Meio, *FA* Fazenda Alegria, *FI* Fazenda Ipiranga, *FR* Fazenda Retirinho, *FZ* Fazenda Zelândia, *MA* Mariante, *MC* Macacoari, *MI* Mimoso, *PF* Porto da Fazenda, *PG*, Pântano Grande, *PR* Praialzinho, *RP* Rio Piquiri, *RV* Rio Vermelho, *SC* Se Cria, *SE* Serrinha, *SM* Santa Maria, *TC* Tucum

The prevalence of *Plasmodium* within each species demonstrated different patterns across regions. For the roseate spoonbill, significant differences were found between populations in the northern and central-western regions (*P* < 0.0001). However, no differences were detected between samples of the great egret in the central-western and southern regions or between wood stork samples in the northern and central-western regions. In comparisons among species within each region, significant differences in prevalence were found between the roseate spoonbill and both the wood stork (*P* < 0.0001) and great egret (*P* < 0.0001), whereas no significant difference was found between the great egret and wood stork samples in the same region (central-western).

As *Haemoproteus* was detected at low levels only in the roseate spoonbill and wood stork, it was not possible to perform statistical tests comparing the parasite prevalences in these two species.

## Discussion

To the best of our knowledge, this is the first report of *Plasmodium* in birds of the family Threskiornthidae, found in natural populations of the roseate spoonbill. *Haemoproteus* has been reported in captive birds of two species of this family, the scarlet ibis (*Eudocimus ruber*) and the southern bald ibis (*Geronticus calvus*) [12, 13]. Telford and colleagues [57] analysed blood smears from 51 nestlings of the roseate spoonbill sampled in Florida (USA), but none were positive based on the morphological test. We report *Plasmodium* in the great egret from the family Ardeidae to amplify the group of published data found in this family: the great blue heron (*Ardea herodias*) [58]; black-crowned night heron (*Nycticorax nycticorax*) [12]; agami heron (*Agamia agami*); striated heron (*Butorides striatus*) [59]; grey heron (*Ardea cinerea*); great bittern (*Botaurus stellaris*) [13]; black bittern (*Ixobrychus flavicollis*) [60]; cattle egret (*Bubulcus ibis*) and little egret (*Egretta garzetta*) [14]; and little bittern (*Ixobrychus minutus*) [61]. There are also reports of ardeids infected by *Haemoproteus* such as the black-headed heron (*Ardea melanocephala*) and cattle egret [14] and by *Leucocytozoon* such as the Japanese night heron (*Gorsachius goisagi*) [62]. In the family Ciconidae, *Plasmodium* and *Haemoproteus* have been reported in the wood stork [10] and *Plasmodium* has been found in the white stork [14].

The nucleotide divergence between clades I and II exceeded the threshold of intraspecific diversity estimated by Perkins [51] for haemosporidians, although this threshold was not exceeded within clade II. These data indicate that clade II represents a species of *Plasmodium* and we have recent evidence for this proposal. Seven wood stork haplotypes (MYCAMP1-7) of clade II were joined into a monophyletic clade with *Plasmodium paranucleophilum* sequences that were isolated and morphologically characterized in six species of birds of prey from the families Accipitriformes, Falconiformes and Strigiformes in southeastern Brazil [63]. This previous study and the composition of clade II with all new lineages herein indicate that *P. paranucleophilum* has low specificity toward bird hosts and it is a generalist. The network structure in clades I and II also presented differences. The comparative analysis of genetic divergence between *Plasmodium* lineages found in the waterbirds studied and the MalAvi database revealed that the lineages described in the present study are genetically distant from others described in the world, confirming a restricted distribution of *P. paranucleophilum*, as proposed by Tostes and colleagues [63]. According to Avise [64], a star-like structure in a network of haplotypes denotes the recent divergence of groups (new haplotypes) from a large ancestral group (common haplotype). Thus, the star-like structure found in clade II of the haplotype network in the present study represents relationships between the *Plasmodium* haplotypes found in the host waterbirds. This structure indicates that the most common haplotype (MYCAMP2) from which the other haplotypes are derived likely represents an ancestral lineage with the ability to infect the waterbird species sampled and therefore has a remarkable ability to spread. We found the MYCAMP2 haplotype in the great egret, roseate spoonbill and wood stork, which may be explained by the fact that these birds breed in mixed colonies and are very gregarious, flying in flocks. The great egret nests in mixed-species or monospecific colonies [65, 66], whereas the roseate spoonbill and wood stork only breed in mixed colonies, nesting with the cattle egret, little blue heron (*Egretta caerulea*), reddish egret (*Egretta rufescens*), snowy egret (*Egretta thula*), tricolored heron (*Egretta tricolor*), great blue heron, black-crowned night-heron, white ibis (*Eudocimus albus*), white-faced ibis (*Plegadis chihi*), glossy ibis (*Plegadis falcinellus*) and olivaceous cormorant (*Phalacrocorax olivaceus*) [67–69]. In homogeneous heronries, the great egret stratifies vertically, occupying a higher nesting niche [66]. When these three species are breeding together, the great egret occupies high nests, the roseate spoonbill seems to be at the bottom and in the middle of the mixed-species hierarchy in nesting colonies [67], and the wood stork predominates in the medium and high nests [69]. In a study conducted in a tropical region, Moens & Pérez-Tris [15] found that *Plasmodium* and *Haemoproteus* communities are more generalist than in temperate areas and we may suppose a similar pattern for *Plasmodium paranucleophilum*, as demonstrated herein and proposed by Tostes and colleagues [63]. We also found new lineages (haplotypes) unique to each species of waterbird and differing from the ancestral lineage by singletons or doubletons, suggesting a recent expansion of this clade of parasites found in waterbird species. Our data agree with findings described in previous studies, which showed a high richness of *Plasmodium* lineages in South America [70, 71], a pattern that had been proposed to be the result of an exceptional radiation along avian hosts in the region [72].

Several studies have focused on the detection of haemosporodians in birds and the spatial distribution of blood parasites, but ecological factors, such as vector abundance, has been insufficiently explored, despite being considered an important factor [73]. The present investigation contributes information on vector abundance using an indirect ecological niche modelling approach that has been useful to understanding the potential distribution of the vector species, such as in the case of the transmission of cutaneous leishmaniasis [74]. Behind this proposal is the assumption that more suitable niches imply a greater number of vectors, which would consequently result in a greater diversity and prevalence of haemosporidians. The sampling sites from which nestling blood was collected were chosen to include representative wetlands located at different latitudes with varied rainfall patterns, diverse vegetation community structures, and different avifauna composition. We found similar habitat suitability for vectors in different wetlands and this result may reflect the greater importance of microhabitats in wetlands to vectors than the macrohabitat of these areas. Several microclimate characteristics need to be examined collectively because they promote the coexistence and diversity of vectors and hosts across geographical areas [75]. Indeed, the availability and characteristics of aquatic habitats are determinants for the breeding of vectors because the preferences of these vectors are species-specific. Some vectors prefer to breed in permanent larval sites, whereas others prefer more transient water sources [76, 77]. The choice of breeding place may differ if the site is exposed to sunlight or under the tree canopy, where water temperatures are lower. Moreover, rainfall and evaporation are equally important to maintaining the viability of sites [78]. Wind direction also exerts an influence by carrying olfactory cues from hosts to female mosquitoes [79].

Supporting our hypothesis, parasite diversity indices were similar for waterbirds sampled in different wetlands, which is in agreement with the similar suitable habitats found on the map and dendrogram for the wetlands studied. The only difference found in haemosporidian diversity for the roseate spoonbill may be the result of the small sample size of the northern population or it may be attributed to a host characteristic or its interaction with other hosts. Host-host interactions in the Pantanal wetland are favoured during the reproduction period of waterbirds. Such interactions are common in the host community in this area, which is an important reservoir of biodiversity in central-western Brazil. The breeding sites and surrounding areas unite a large variety of bird species that use the region as a breeding site, place of residence or stopover area [24]. In an evaluation of bird species richness and the ratio of seasonal to resident bird species in an aquatic habitat of the Pantanal wetland, Figueira and colleagues [80] found that seasonal species comprised 46 to 54% of the 60 species counted and that migration increased during the dry season, which is when waterbird breeding sites were sampled in the present study. The high level of host generalization for *Plasmodium paranucleophilum* [63] in a highly diverse host community, as that found in the Pantanal region, may have resulted in a greater diversity of haemosporidian lineages, as observed in this study in the roseate spoonbill.

The pattern of habitat suitability for vectors on the map also did not explain the differences regarding the prevalence of parasites in the roseate spoonbill between the central-western and northern regions. We supposed that the high prevalence found in this bird species in the central-western region (74%) may be explained by host characteristic or ecological heterogeneities other than vector abundance. Among three regions sampled, the central-western region has a medium mean temperature during the breeding period (26.7 °C) and a medium precipitation index (57.1 mm) [23]. Thus, the hypothesis that higher prevalence in the roseate spoonbill is associated with higher temperatures and precipitation in the region from which the blood samples were taken, is not supported by the present data.

Comparisons within the central-western wetland revealed higher levels of infection in the roseate spoonbill compared to the great egret and wood stork. Removing the environmental effect in this case, we considered characteristics of the hosts. The difference may be partially attributed to the difference in the development of plumage among these species. Roseate spoonbill and wood stork nestlings remain a longer period with exposed skin than great egret nestlings, making both species more susceptible to mosquito bites [65, 68, 69]. Another host factor regards migrant or non-migrant characteristics. Migration is pronounced among aquatic birds: 35% of species that use freshwater habitats and reproduce in the Neotropics are migratory and even many resident species regularly move in response to fluctuations in water level or resource availability [81]. While the great egret is a resident in the wetlands sampled, the roseate spoonbill and wood stork are migratory and are therefore exposed to several parasite faunas during movements between breeding sites and stopover areas, as observed in the willow warbler [75]. Wood storks and roseate spoonbills move from breeding to post-breeding sites, covering distances as long as 3900 km from tropical and subtropical regions to temperate regions [82, 83]. During these movements, they carry their own infections and are exposed to a greater variety of pathogens. Thus, they can spread pathogens in these regions and infect resident species such as great egret. An extensive analysis involving the haemosporidian parasite fauna of resident and migratory birds [84] showed that the degree of parasite lineage sharing between migrants and residents in breeding and wintering areas appears to reflect, to a large degree, the taxonomic similarity of migrants to the resident species in both areas. The phylogenetic component was also found in the present data, which showed MYCAMP2 to be the most common lineage in two migrants and one resident waterbird species that are taxonomically related. Migratory status is reported to have also a significant effect on the prevalence of *Haemoproteus* in passerines in Spain [85]. However, plumage timing and migratory status alone do not explain the higher prevalence of haemosporidians in the roseate spoonbill in comparison to the great egret and wood stork. Thus, other variables, such as immune status, should be tested in future studies.

## Conclusions

The map of habitat suitability for avian vectors is a useful contribution for understanding the distribution of blood parasites involving different bird groups in different regions of Brazil. We found that wetlands located in a 30° latitudinal range offer similar conditions for vectors, which is a pattern that agrees with similar parasite diversity indices found for waterbirds in these areas. The present study offers evidence of a new group of birds infected by *Plasmodium paranucleophilum*. The approach of sampling the same host species in different landscapes was tested and enabled inferences regarding the effects of environmental and host characteristics on the diversity and prevalence of haemosporidians.

## Additional files

Additional file 1: **Table S1** Geographical coordinates of sampled sites where blood samples were collected from waterbirds nestlings and the suitability of habitat for avian haemosporidians vectors. **Table S2** Summary information about MalAvi lineages included in the network analysis. (DOCX 23 kb)

Additional file 2: **Figure S1** Dendrogram showing relations defined by the suitability of habitat for vectors among sites where blood samples were collected. Codes of waterbird colonies are described in Additional file 1: Table S1; regions are indicated by colors. (TIF 502 kb)

Additional file 3: **Figure S2** Nucleotide diversity, haplotype diversity and their variances for great egret (GE), roseate spoonbill (RS) and wood stork (WS), in samples from wetlands located in three regions of Brazil. (TIFF 2700 kb)

## Abbreviations

PCR: Polymerase chain reaction
GBIF: Global Biodiversity Information Facility
*cytb*: Cytochome b
*Plas*: *Plasmodium* spp.
*Haem*: *Haemoproteus* spp.
