# Supplementary material for: Effects of habitat suitability for vectors, environmental factors and host characteristics on the spatial distribution of the diversity and prevalence of haemosporidians in waterbirds from three Brazilian wetlands
Source: Parasit Vectors. 2018 May 2;11:276. doi: 10.1186/s13071-018-2847-z (PMC5930942; doi:10.1186/s13071-018-2847-z)
Supplement: Supplementary file 1 — Table S1 Geographical coordinates of sampled sites where blood samples were collected from waterbirds nestlings and the suitability of habitat for avian haemosporidians vectors. Table S2 Summary information about MalAvi lineages included in the network analysis. (DOCX 23 kb) [file 13071_2018_2847_MOESM1_ESM.docx]

**Additional file 1**

**Additional file 1: Table S1.** Geographical coordinates of sampled sites where blood samples were collected from waterbirds nestlings and the suitability of habitat for avian haemosporidians vectors.

| Species | Geographical region within Brazil | Geographical coordinates | Breeding colony | Code | N | Suitability habitat |
| --- | --- | --- | --- | --- | --- | --- |
| Great egret | Center-West | 16°26’S 56°03’W | Tucum | TU | 8 | 0.5366320 |
|  |  | 16°28’S 56°07’W | Porto da Fazenda | PF | 15 | 0.5324490 |
|  |  | 16°46’S 56°35’W | Praialzinho | PR | 19 | 0.5074810 |
|  |  | 16°18’S 57°02’W | Campo do Meio | CM | 2 | 0.4871410 |
|  | South | 29°42’S 53°16’W | Santa Maria | SM | 33 | 0.5404260 |
|  |  | 30°22’S 54°04’W | Serrinha | SE | 20 | 0.5403740 |
|  |  | 29°40’S 51°58’W | Mariante | MA | 22 | 0.6226980 |
|  |  | 30°10’S 52°20’W | Pântano Grande | PG | 12 | 0.5751140 |
| Roseate spoonbill | North | 01°56’N 50°35’W | Se Cria | SC | 17 | 0.7506420 |
|  |  | 01°09'N 50°23'W | Fazenda Zelândia | FZ | 11 | 0.7175610 |
|  | Center-West | 17°07’S 56°21’W | Rio Piquiri | RP | 2 | 0.5076700 |
|  |  | 16°09’S 55°48’W | Mimoso | MI | 1 | 0.5345650 |
|  |  | 19°50’S 56˚02´W | Fazenda Retirinho | FR | 10 | 0.5287500 |
|  |  | 16°25’S 56°36´W | Fazenda Ipiranga | FI | 2 | 0.5973348 |
|  |  | 16^o^39’S 57^o^10’W | Baía de Gaíva | BG | 6 | 0.4847000 |
|  |  | 16°46’S 56°35’W | Praialzinho | PR | 2 | 0.5074810 |
| Wood stork | North | 01°56’N 50°35’W | Se Cria | SC | 20 | 0.7506420 |
|  |  | 01°02’N 50°32’W | Fazenda Alegria | FA | 9 | 0.5335481 |
|  |  | 01°09'N 50°23'W | Fazenda Zelândia | FZ | 16 | 0.7175610 |
|  |  | 00°27’N 50°40’W | Macacoari | MC | 29 | 0.6233570 |
|  | Center-West | 16°25’S 56°36´W | Fazenda Ipiranga | FI | 15 | 0.5973348 |
|  |  | 16^o^39’S 57^o^10’W | Baía de Gaíva | BG | 22 | 0.4847000 |
|  |  | 16°26’S 56°03’W | Tucum | TU | 16 | 0.5366320 |
|  |  | 16°28’S 56°07’W | Porto da Fazenda | PF | 25 | 0.5324490 |
|  |  | 19°50’S 56˚02´W | Fazenda Retirinho | FR | 16 | 0.5287500 |
|  |  | 18°40'S 56°25'W | Baía Bonita | BB | 16 | 0.5973348 |
|  |  | 19°36'S 56°51'W | Rio Vermelho | RV | 15 | 0.5701238 |

**Additional file 1:** **Table S2.** Summary information about MalAvi lineages included in the network analysis.

| Lineage | Similarity | Dif bases | Locality | Host Family | Host species | | Genbank | | Ref | |
| --- | --- | --- | --- | --- | --- | --- | --- | --- | --- | --- |
| RAMCAR04 | 97 | 11-13 | Brazil (AM) | Fringillidae | *Ramphocelus carbo* | | KU562678 | | Fecchio et al., 2017 | |
| PSABIF01 | 97 | 11-13 | Brazil (AM) | Icteridae | *Psarocolius bifasciatus* | | KU562734 | | Fecchio et al., 2017 | |
| BAFLA03 | 97 | 11-14 | Americas | Several Orders | Lots of species | | JX021476 | | Lacorte et al., 2013 | |
| PAPOL06 | 97 | 11-14 | Brazil (MG) | Tyrannidae | *Pachyramphus polychopterus* | | JX029888 | | Lacorte et al., 2013 | |
| TACCRI02 | 97 | 11-14 | Brazil (AM) | Fringillidae | *Tachyphonus cristatus* | | KU562448 | | Fecchio et al., 2017 | |
| MYRAXI08 | 97 | 12-14 | Brazil (AM) | Thamnophilidae | *Myrmotherula axillaris* | | KU562675 | | Fecchio et al., 2017 | |
| RAMCAR05 | 97 | 12-15 | Brazil (AM) | Fringillidae | *Ramphocelus carbo* | | KU562679 | | Fecchio et al., 2017 | |
| PARCAP06 | 97 | 12-15 | Brazil (MS) | Fringillidae | *Paroaria capitata* | | KU562689 | | Fecchio et al., 2017 | |
| NYSCHA02 | 97 | 12-15 | Brazil (DF) | Bucconidae | *Nystalus chacuru* | | KU562553 | | Fecchio et al., 2017 | |
| ARRTAC01 | 97 | 12-15 | Brazil (PA) | Fringillidae | *Arremon taciturnus* | | KU562438 | | Fecchio et al., 2017 | |
| SCHTUR02 | 97 | 13-16 | Brazil (PA) | Pipridae | *Schiffornis turdina* | | KU562434 | | Fecchio et al., 2017 | |
| PHEGEN01 | 97 | 13-16 | Peru | Certhiidae | *Pheugopedius genibarbis* | | KU562811 | | Fecchio et al., 2017 | |
| PARCAP07 | 97 | 13-16 | Brazil (DF) | Fringillidae | *Paroaria capitata* | | KU562690 | | Fecchio et al., 2017 | |
| HEMGRI01 | 97 | 13-16 | Peru | Tyrannidae | *Hemitriccus griseipectus* | | KU562834 | | Fecchio et al., 2017 | |
| AUTINF02 | 97 | 15 | Brazil (RO) | Furnariidae | *Automolus infuscatus* | | KU562603 | | Fecchio et al., 2017 | |
|  |  |  |  |  |  |  | |  | |  |

**References**

Fecchio A, Pinheiro R, Felix G, Faria IP, Pinho JB, Lacorte GA, et al. Host community similarity and geography shape the diversity and distribution of haemosporidian parasites in Amazonian birds. Ecography. 2017;41:505–15.

Lacorte GA, Felix GM, Pinheiro RR, Chaves AV, Almeida-Neto G, Neves FS, et al. Exploring the diversity and distribution of Neotropical avian malaria parasites - a molecular survey from Southeast Brazil. PLoS One. 2013;8:e57770
