# Supplementary figures and images for: Effects of habitat suitability for vectors, environmental factors and host characteristics on the spatial distribution of the diversity and prevalence of haemosporidians in waterbirds from three Brazilian wetlands
Source: Parasit Vectors. 2018 May 2;11:276. doi: 10.1186/s13071-018-2847-z (PMC5930942; doi:10.1186/s13071-018-2847-z)

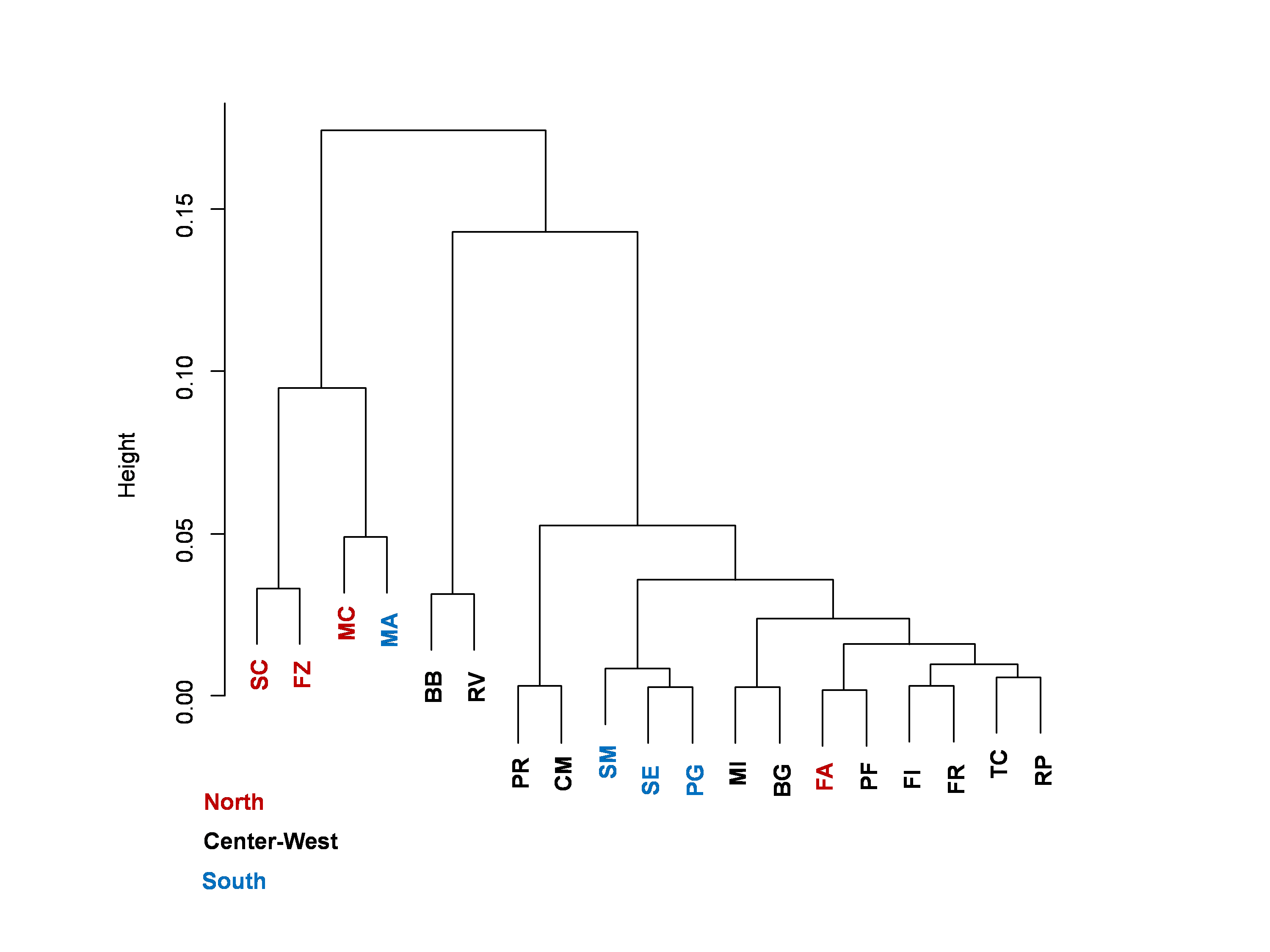

Supplement: Supplementary file 2 — Figure S1 Dendrogram showing relations defined by the suitability of habitat for vectors among sites where blood samples were collected. Codes of waterbird colonies are described in Additional file 1: Table S1; regions are indicated by colors. (TIF 502 kb) [file 13071_2018_2847_MOESM2_ESM.tif]

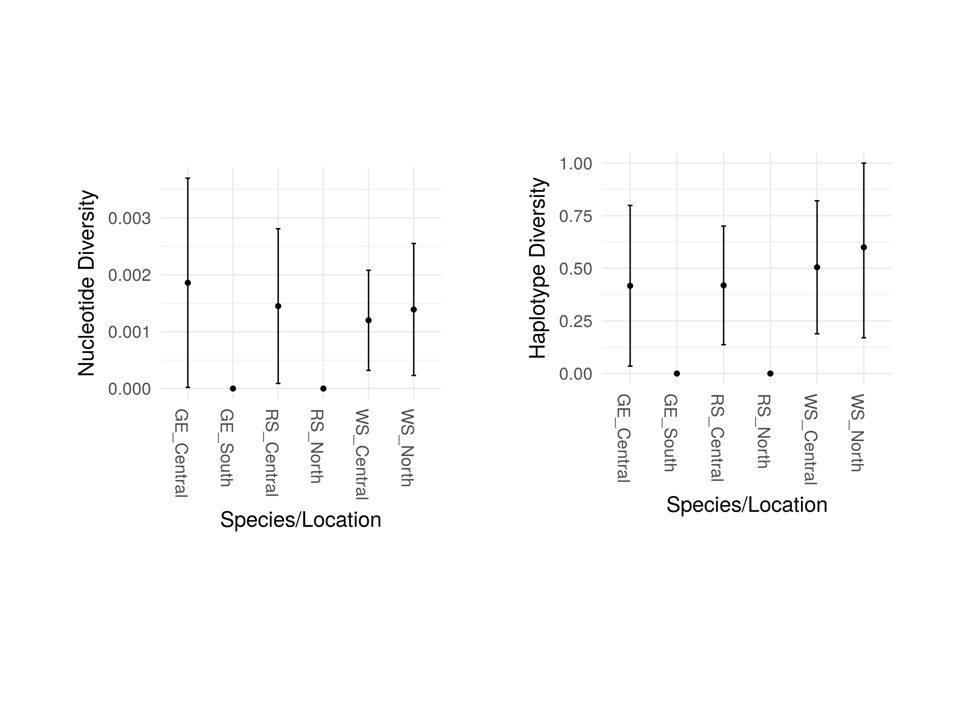

Supplement: Supplementary file 3 — Figure S2 Nucleotide diversity, haplotype diversity and their variances for great egret (GE), roseate spoonbill (RS) and wood stork (WS), in samples from wetlands located in three regions of Brazil. (TIFF 2700 kb) [file 13071_2018_2847_MOESM3_ESM.tiff]
